# Identification of the MAPK Cascade and its Relationship with Nitrogen Metabolism in the Green Alga *Chlamydomonas reinhardtii*

**DOI:** 10.3390/ijms21103417

**Published:** 2020-05-12

**Authors:** Aitor Gomez-Osuna, Victoria Calatrava, Aurora Galvan, Emilio Fernandez, Angel Llamas

**Affiliations:** Departamento. de Bioquímica y Biología Molecular, Campus de Rabanales y Campus Internacional de Excelencia Agroalimentario (CeiA3), Edificio Severo Ochoa, Universidad de Córdoba, 14071 Córdoba, Spain; b12goosa@uco.es (A.G.-O.); calatravavictoria@gmail.com (V.C.); bb1gacea@uco.es (A.G.); bb1feree@uco.es (E.F.)

**Keywords:** MAPK, MAPKK, MAPKKK, nitrogen, Chlamydomonas, nitratereductase, nitric oxide

## Abstract

The mitogen activated protein kinases (MAPKs) form part of a signaling cascade through phosphorylation reactions conserved in all eukaryotic organisms. The MAPK cascades are mainly composed by three proteins, MAPKKKs, MAPKKs and MAPKs. Some signals induce MAPKKK-mediated phosphorylation and activation of MAPKK that phosphorylate and activate MAPK. Afterward, MAPKs can act either in the cytoplasm or be imported into the nucleus to activate other proteins or transcription factors. In the green microalga *Chlamydomonas reinhardtii* the pathway for nitrogen (N) assimilation is well characterized, yet its regulation still has many unknown features. Nitric oxide (NO) is a fundamental signal molecule for N regulation, where nitrate reductase (NR) plays a central role in its synthesis. The MAPK cascades could be regulating N assimilation, since it has been described that the phosphorylation of NR by MAPK6 promotes NO production in *Arabidopsis thaliana*. We have identified the proteins involved in the MAPK cascades in *Chlamydomonas reinhardtii*, finding 17 MAPKs, 2 MAPKKs and 108 MAPKKKs (11 MEKK-, 94 RAF- and 3 ZIK-type) that have been structurally and phylogenetically characterized. The genetic expressions of MAPKs and the MAPKK were slightly regulated by N. However, the genetic expressions of MAPKKKs RAF14 and RAF79 showed a very strong repression by ammonium, which suggests that they may have a key role in the regulation of N assimilation, encouraging to further analyze in detail the role of MAPK cascades in the regulation of N metabolism.

## 1. Introduction

*Chlamydomonas reinhardtii* (Chlamydomonas) has been used as a model organism since the mid-last century, thanks to its properties as a single-celled, photosynthetic and haploid organism, with a short doubling time (8 h), with both sexual or asexual reproduction and having at the molecular level many of the main characteristics of higher plants [1]. In recent years, the development of transformation techniques and the sequencing of its genome have remarkably enhanced its use in research as a model for both plants and animals [2]. In addition, the easy manipulation of its genome has allowed the creation of a collection of about 60,000 mutants molecularly tagged [3]. As such a model system, it has promoted scientific advances in a wide variety of fields, such as photosynthesis [4], flagellar motility [5], sulfur metabolism [6], hydrogen production [7], and N metabolism [8].

N is one of the essential elements for all living beings. Interestingly, one of the most important N sources is atmospheric N_2_, however, it can only be assimilated by some prokaryotic organisms and with a high energetic cost [9]. In photosynthetic organisms, the lack of N in easily assimilable forms, mainly ammonium (NH_4_^+^) and nitrate (NO_3_^−^), is a limiting factor for their growth, affecting seriously agricultural production [10]. Therefore, to increase crop yields, for years, large amounts of N fertilizers have been abusively used in many cases, which has had a strong environmental and public health impact [11]. For nitrate, it is estimated that only one third of the total amount present in soils is assimilated by crops, while the rest is lost by leaching, which results in continental and oceanic waters eutrophication and the consequent environmental problems that this entails [12]. Therefore, a detailed understanding of N assimilation and its regulation is essential.

In plants and algae N metabolism has been well described and reviewed [10,13,14,15,16]. In addition, in Chlamydomonas the N assimilation has also been widely studied. Chlamydomonas can incorporate different N sources, organic as urea and amino acids or inorganic as ammonium, nitrate or nitrite. Concerning nitrate assimilation, it is first transported into the cytoplasm by specific transporters (NRT1 and NRT2). Then, nitrate reductase (NR) reduces nitrate to nitrite. Subsequently, nitrite is transported to the chloroplast by NAR1 transporters [17]. Then nitrite reductase (NIR) catalyzes nitrite reduction to ammonium, which is finally incorporated into amino acids by the GS/GOGAT pathway [8]. In Chlamydomonas, nitrate itself is the signal for activating its assimilation, inducing the main positive regulatory gene involved in nitrate assimilation, the transcription factor NIT2. In contrast, ammonium is an immediate repressor signal for nitrate assimilation, inhibiting NIT2 [18]. However, many important questions remain unresolved in the regulation of N assimilation. For example, how NIT2 detects the absence of ammonium and gets activated.

The NR is a 100–120 kDa homodimeric enzyme conserved in eukaryotic organisms. In plants there can be up to two NR genes (*NIA1* and *NIA2*) as it occurs in *Arabidopsis thaliana* (Arabidopsis). On the other hand, in fungi and algae, there is generally one gene, as in Chlamydomonas (*NIA1*). Each NR subunit contains three prosthetic groups, FAD, cytochrome b5 (heme) and molybdenum cofactor (Moco) which form three different functional domains separated by two hinge regions [19]. Nitric oxide (NO) has been widely described in many organisms as a signal molecule in various processes [20]. In Chlamydomonas, NO has been related to negative regulation of nitrate assimilation [21], N and S deficiency stresses [22], chloroplast biogenesis [23], programmed cell death [24], responses to darkness and hypoxia [25] and saline stress [26]. Interestingly, in addition to its role in N assimilation, reducing nitrate to nitrite, NR also plays a fundamental regulatory role in N assimilation by its capacity to produce and regulate the NO levels. The FAD and heme domains of NR can participate in different types of reactions, previously proposed to provide electrons to the Moco domain to convert nitrate into nitrite, and then to directly reduce nitrite to NO [27,28,29]. However, the physiological function of this nitrite reducing activity is very controversial, since NR has a much lower Km for nitrate (10 µM) than for nitrite (100 µM) and in addition, nitrate strongly inhibits the reaction (Ki = 50 µM). It is estimated that only 1% of NR activity in vitro is associated with the production of NO [30]. Recently, another way of in which NR is involved in the regulation of NO levels is by incorporating accessory proteins (indirect reduction) [15,31]. One of these accessory proteins is NOFNiR, a molybdoenzyme that only contains a Moco domain. NR provides electrons to NOFNiR, where nitrite is reduced to NO. Also, NR can provide electrons to a truncated hemoglobin, THB1, catalyzing the oxidation of NO to nitrate (NO consumption). These three reactions, NO_3_^−^→NO_2_^−^→NO→NO_3_^−^, make up the NO cycle, regulated by NR [32]. However, the mechanisms that govern NOFNiR-NR and THB1-NR interaction in the regulation of NO levels remain unknown.

The main function for MAPK cascades is to integrate external stimuli into the cells to trigger a response. In this way, MAPK cascades have been related to a wide range of processes that include responses to biotic and abiotic stresses, development, growth and hormonal signaling [33]. The MAPK cascade involves a series of successive reversible phosphorylation reactions of three types of protein kinases: MAPK, MAPK kinase (MAPKK) and MAPKK kinase (MAPKKK). First, MAPKKK gets activated by phosphorylation, which can happen through membrane receptor proteins such as other kinase, GTPases or other stimuli [34]. In turn, activated MAPKKK phosphorylate MAPKK in the activation motif promoting their activation. This activation sites consist of two serine or threonine residues separated by five amino acid residues in plants and three in animals [35]. On the other hand, activated MAPKK phosphorylate MAPK in threonine and tyrosine residues at the activation motif of sequence TxY. The MAPKs are the final effectors of signal transmission, that is, they can act either in the cytoplasm or in the nucleus and to phosphorylate other enzymes, kinases or transcription factors among others [36]. MAPKs phosphorylate proteins in a serine or threonine position directly followed by a proline (S/TP motif). In Arabidopsis, 20 MAPKs, 10 MAPKKs and 80 MAPKKKs; in corn, 19 MAPKs, nine MAPKKs and 74 MAPKKKs and in rice 17 MAPKs, eight MAPKKs and 75 MAPKKKs have been described [37]. Similar proportions are also present in other organisms which implies a convergence of the MAPKKKs over the MAPKKs and, in turn, a divergence of MAPKKs over MAPKs [38].

In recent years, knowledge on MAPK cascade has increased significantly, however, only a few MAPK cascades have been fully described, partly due to the lack of characterization and attention paid to MAPKKKs [39]. To date, the function of most MAPKKKs is unknown and it is even suspected that some of them may perform additional functions in addition to intervening in the MAPK signaling cascade [40]. Some studies have established a relationship between components of the MAPK cascade and the N metabolism. In Arabidopsis it has been established that the exposure to high concentrations of H_2_O_2_ induces the production of NO by NR. Interestingly, this NO production is regulated by a signaling cascade mediated by MAPK6 [41]. The authors observed that H_2_O_2_ activates a MAPK signaling cascade in which MAPK6 phosphorylates NR2 and not NR1, resulting in an increase in NO production that enhances the lateral root growth. On the other hand, NR1 did not show any interaction with MAPK6 in vivo, but only in vitro [41]. In this sense, the simple *mapk6* and *nia2* mutants, and the double mutant, did suffer a decrease in NO production during exposure to H_2_O_2_. Nevertheless, important questions remain to be resolved, such as which MAPKK activates MAPK6.

Recently, some MAPKs [42,43,44] or MAPKKs [35,45,46] have been identified in Chlamydomonas and partly characterized. However, to date a systematic and extensive search of the whole MAPK cascade has not been conducted. The main objective of this work is to discover in Chlamydomonas whether the MAPK cascade has any relation with N metabolism regulation. In this work, each component of the MAPK families of Chlamydomonas is structurally and phylogenetically identified and characterized, and then some of the MAPK candidates from each family are studied and shown to have a possible role in the regulation of N assimilation.

## 2. Results

### 2.1. Chlamydomonas MAPK Family

The search for Chlamydomonas MAPKs members was performed by using the conserved sequence of the activation domain TxYVxTRWYRAPE (L/V) [37]. This search generated 17 MAPKs orthologs. In the Chlamydomonas genome seven MAPKs were already annotated: MAPK2, MAPK3, MAPK4, MAPK5, MAPK6, MAPK7 and MAPK8 and we have respected that annotation. The annotation of the other 10 MAPKs is done here for the first time. Table S1 summarizes the main characteristics of these 17 MAPKs. The exons number was in between seven (MAPK7) and 12 (MAPK2 and MAPK10), the amino acid residues number ranged from 394 (MAPK6) to 2054 (MAPK1), and the molecular masses ranged from 42.52 kDa and 209.92 kDa, respectively. The theoretical isoelectric point (pI) was between 4.53 (MAPK1) and 9.91 (MAPK9), the gene length from 2446 nt (MAPK3) to 10,268 nt (MAPK10). As for the predicted subcellular location, most of them went to the nucleus, with the exception of MAPK3, MAPK5, MAPK6 and MAPK8, whose predicted location was ambiguous and could also be located in the cytoplasm or in the mitochondria.

**Figure 1:**
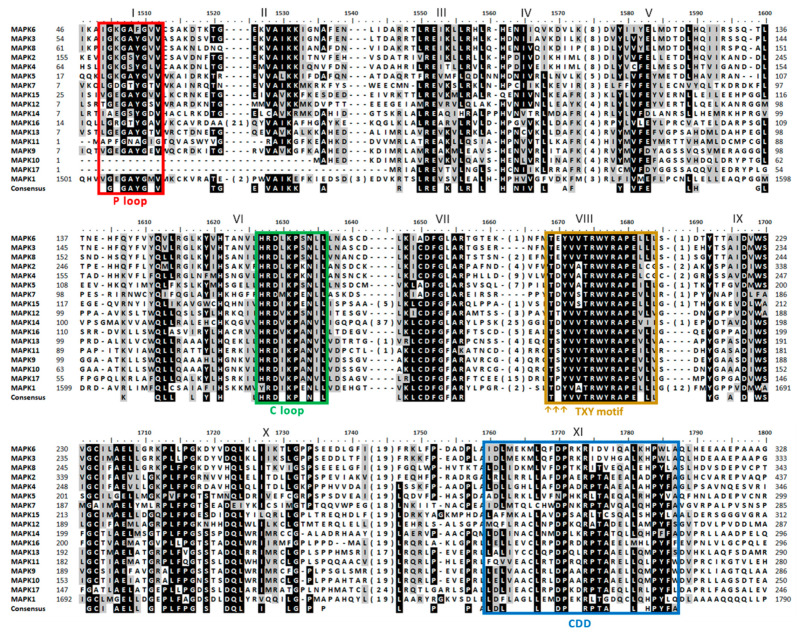
Sequence alignment of MAPK family. A simplified alignment (ClustalW) of the 17 MAPKs identified along with the consensus sequence is shown. The 11 MAPK domains are shown in roman numerals (I-XI). The TxY motif is highlighted with three arrows (↑) in the activation domain. Highly conserved amino acids are shown on a black background, and moderately conserved amino acids are shown on a gray background. The numbers in brackets in the alignment represent the lengths of poorly conserved inserts that have not been shown to maximize the alignment. The P, C and T loops and the CDD are highlighted in colored rectangles.

The alignment of the 17 MAPKs showed a total of 11 conserved domains (Figure 1). The most characteristic MAPK domain is TxY (T loop) which is the key activation domain present in all MAPKs [33]. As shown here, all the 17 MAPKs had the TxY motif (Figure 1, domain VIII). The TxY motifs identified were mostly TEY (MAPK3, 6, 8) and TDY (MAPK1, 2, 4, 5, 7, 14, 15), similarly to what has been previously found in land plants [47]. Other MAPK motif was the phosphate binding site (P loop) that had the conserved sequence IGxGxxGxV located at the N-terminal domain [47] (Figure 1, domain I and Table S2). It is noteworthy that MAPK10 and MAPK17 lacked this P loop. Another motif was the catalytic site (C loop) that was located near the N-terminal domain, showing the conserved sequence HRD(L/I/V)KPxN [47] (Figure 1, domain VI and Table S2). The consensus docking domain (CDD) is the one that interacts with the proteins that are going to be phosphorylated and gives the specificity for a particular substrate binding. In this sense, CDD has a key role in recognition and substrate binding [48]. In all the Chlamydomonas MAPKs, the CDD was located at the C-terminal domain, and different sequences are revealed depending on the MAPK (Figure 1, domain XI and Table S2). Indeed, the CDD has a variable sequence in all organisms and sometimes is used to establish the MAPK phylogenetic classification [35,45,47]. The presence of those domains indicates that it is very likely that the 17 MAPKs identified are actual MAPK.

**Figure 2:**
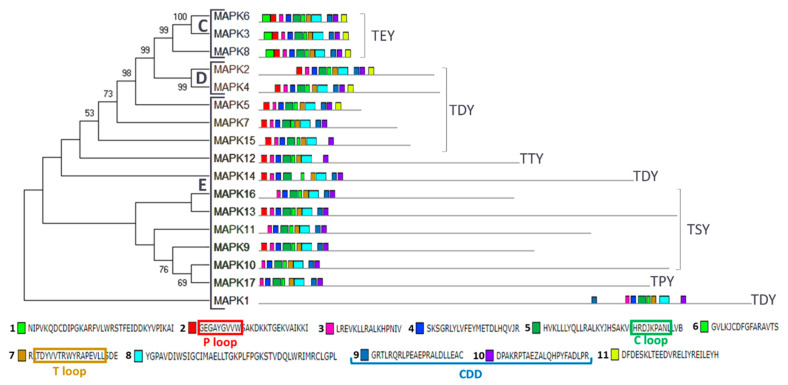
Phylogenetic and motif analysis of MAPK family. The MAPKs classified by maximum likelihood phylogenetic analysis is shown on the left. Numbers on branches indicate percent bootstraps values. Bootstrap values above 50%, based on 1000 replicates, are shown. On the right, the analysis of their domains identified with the MEME program. Below the conserved sequence of the 11 identified domains listed in order of appearance from the N-terminal domain. The P, C and T loops and the CDD are highlighted in colored rectangles. Capital letters indicate the different phylogenetic groups.

In plants, MAPKs are usually classified phylogenetically into four groups (A–D) [33,49]. Our phylogenetic and domain analysis of Chlamydomonas MAPKs revealed the presence of three groups (Figure 2 and Figure S1). We have found three in group C (MAPK3, 6 and 8), two in group D (MAPK2 and 4) and 12 in a new group E (MAPK1, 5, 7, 9–17). Group C is characterized by having TEY in the T loop, a highly conserved CDD and a short C-terminal domain. Group D are MAPKs with TDY in the T loop. New termed group E shows a variety of activation loops and the kinase domain is located at the N-terminal domain, leaving tails of different length except for MAPK1. This protein has preserved the P, C and T loops and the CDD, so it seems a real MAPK, but its kinase domain is located at the C-terminal domain (Figure 2). Phylogenetic analysis with other organisms (Figure S1) also revealed a relation of MAPK5 with the ERK7 subfamily as it was previously described [43].

### 2.2. Chlamydomonas MAPK Gene Expression

**Figure 3:**
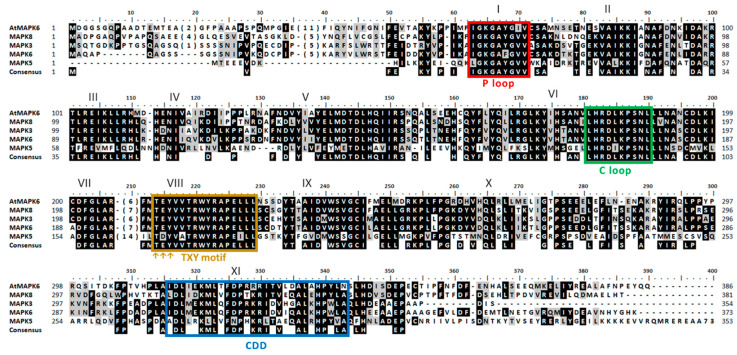
Sequence alignment of Arabidopsis MAPK6 with the most closely related Chlamydomonas MAPK. For other details see Figure 1.

The expression of most genes involved in N assimilation depends on the type of N source present [31]. A gene expression of any of the MAPK inducible or repressible by some N source, would suggest that they could be involved with the assimilation of N. In Arabidopsis MAPK6 phosphorylates the NR regulating the production of NO [41]. Therefore, the genetic expression of the four Chlamydomonas MAPKs showing the highest identity to Arabidopsis MAPK6 (AtMAPK6) was studied. The 17 Chlamydomonas MAPKs were compared with AtMAPK6, the four with the highest identity were MAPK8 (68%), MAPK3 (64%), MAPK6 (61%) and MAPK5 (42%) (Figure 3).

**Figure 4:**
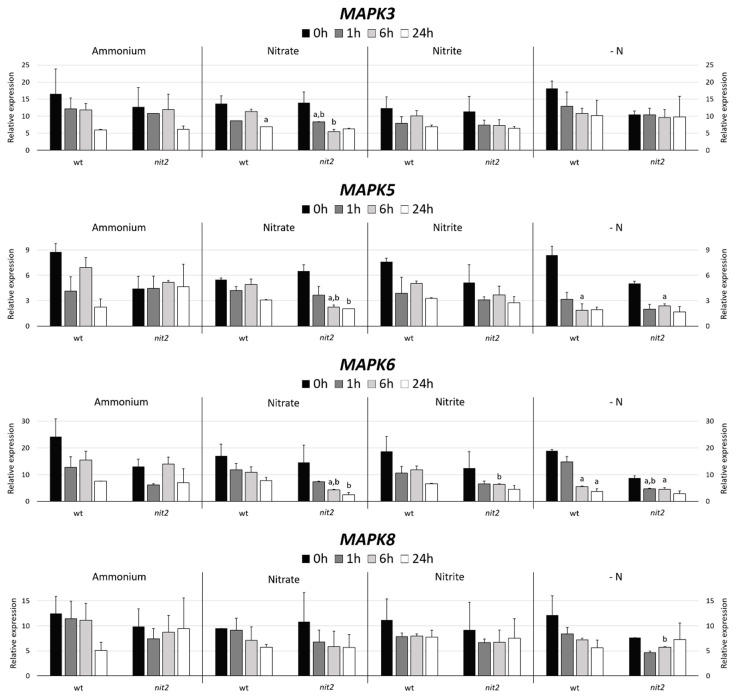
*MAPK* gene expression. The gene expression of Chlamydomonas *MAPK3*, *MAPK5*, *MAPK6* and *MAPK8* were studied in the wt (704) and *nit2* (89.87) at different times, in medium without nitrogen (-N) and with ammonium (8 mM), nitrate (4 mM), or nitrite (2 mM). The gene expression was quantified by RT-qPCR, the value 0 was assigned to the expression level of internal standard gene ubiquitine ligase in each condition. Error bars indicate the standard deviation of three biological replicates. Comparisons were made by the Student’s *t*-test. (a) Indicates statistical differences (*p* < 0.05) compared to ammonium medium in the same strain and (b) compared to the wt strain in the same medium (see *Materials and Methods* section for further explanations).

Two Chlamydomonas strains, a wt (704) and a mutant in the transcription factor NIT2 (*nit2*), which is the main regulator of the expression of genes involved in the N assimilation were used. The *MAPK3* expression decreased in ammonium, nitrate and nitrite with time with no appreciable differences in both strains. However, it is noteworthy, that in media without nitrogen (-N) *nit2* showed a *MAPK3* expression maintained over time. This indicates that *NIT2* may be essential for *MAPK3* to sense the absence of N source (Figure 4). In both strains, the *MAPK5* expression suffered a moderated decrease in nitrate, nitrite and even stronger in -N medium. However, the *nit2* mutant showed a maintained expression of *MAPK5* in ammonium. This might indicate that *MAPK5* needs *NIT2* to sense the presence of ammonium. The *MAPK6* expression was very similar to *MAPK3*, except for the *nit2* mutant in -N media, where its expression was noticeably lower. 

The *MAPK8* expression in ammonium falls strongly after 24 h in the wt. In nitrate and nitrite media, the *MAPK8* expression was very similar in both strains. However, in -N media, the expression in the wt decreased and in *nit2* remains stable, which coincides with *MAPK3*. This suggests that *NIT2* is also essential for *MAPK8* sensing of N absence.

### 2.3. Study of a Chlamydomonas Mapk8 Mutant

Another way to assess whether any of the MAPK is involved in N metabolism is to study the growth phenotype of MAPK mutants in media with different N sources. The Chlamydomonas MAPK8 shows the highest identity to Arabidopsis MAPK6. For this reason, a search in the Chlamydomonas mutant library (https://www.chlamylibrary.org) was performed, and a strain *mapk8* mutant was obtained. A drawback of this mutant library is that the parental strain has mutated the NR (*nia1*) and the transcription factor NIT2 (*nit2*), both essential for the correct assimilation of N. Therefore, a genetic cross was made with the wt strain 21gr. Two strains *mapk8-1* and *mapk8-2*, both *mapk8* mutants but wt for the assimilation of N, were obtained.

**Figure 5:**
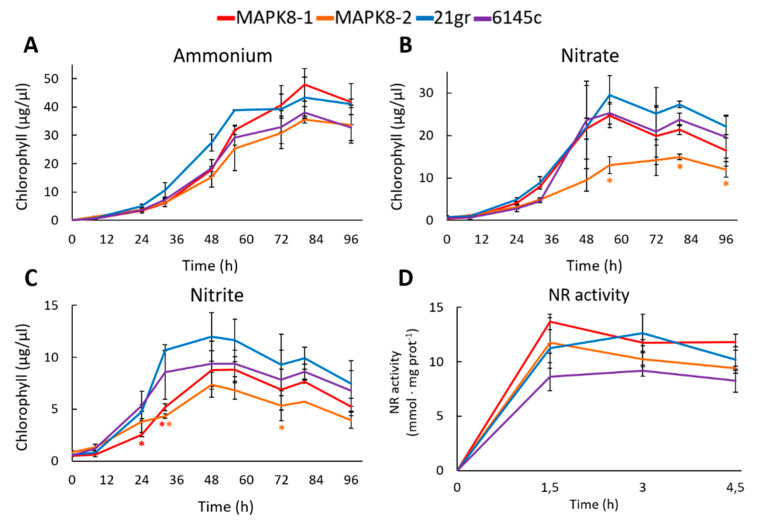
Growth and NR activity in *mapk8* and wt strains. Two *mapk* mutants (*mapk8-1* and *mapk8-2*) and two wt (6145c and 21gr) strains were grown in the following media (**A**) 8 mM ammonium, (**B**) 4 mM nitrate, and (**C**) 2 mM nitrite and at the indicated times samples were taken to quantify the chlorophyll. (**D**) The indicated strains were grown in ammonium until de exponential phase of growth, afterward the cultures were induced with 4 mM nitrate and at the indicated times samples were taken to measure the NR activity. Error bars indicate the standard deviation of three biological replicates. Comparisons were made by the Student’s *t*-test. Statistical differences (* *p* < 0.05) are shown between a *mapk8* mutant and both wt strains of the same time point. The red and orange asterisks represent statistically significant difference with mapk8-1 and mapk8-2 respectively.

Physiological studies were made between the two *mapk8* and two wt strains 21gr and 6145c. First, the growth in media with different N sources was studied. In ammonium, the growth of *mapk8* mutants was very similar to the growth of both wt strains 21gr and 6145c (Figure 5A). In nitrate and nitrite, both *mapk8* mutants grew less, especially *mapk8-2*, than both wt (Figure 5B,C). This would indicate a slightly effect of MAPK8 in nitrate and nitrite assimilation. One reason of the lower growth in nitrate and nitrite may be an alteration in the NR activity. Therefore, the NR activity was determined. As a result, no appreciable differences in NR activity were observed (Figure 5D). Therefore, MAPK8 seem to have a minor effect on NR activity.

### 2.4. Chlamydomonas MAPKK Family

To search Chlamydomonas MAPKKs members the conserved sequence of the activation domain GTxxYMSPER was used [37]. This search generated two MAPKKs, both with the S/TxxxxxS/T motif essential for its activation [50]. In Table S1, its main properties are summarized. In the Phytozome database, both MAPKKs are annotated as MAPKK1. Therefore, they were renamed here as MAPKK1, which is located on chromosome 6, and MAPKK2 that is located on chromosome 13. The gene length of both was similar: MAPKK1 4344 nt and MAPKK2 4286 nt. However, the exons number was much higher for MAPKK1 (14) than for MAPKK2 (7). The protein length was 452 aa and 473 aa, the pI 6.87 and 8.16 and the molecular mass 50.28 kDa and 47.34 kDa, for MAPKK1 and MAPKK2, respectively. The predicted subcellular location was nuclear for MAPKK2 and ambiguous for MAPKK1. However, it is noteworthy that MAPKK1 has an NTF domain (nuclear transfer factor) at its C-terminal domain, so it could be transported to the nucleus.

**Figure 6:**
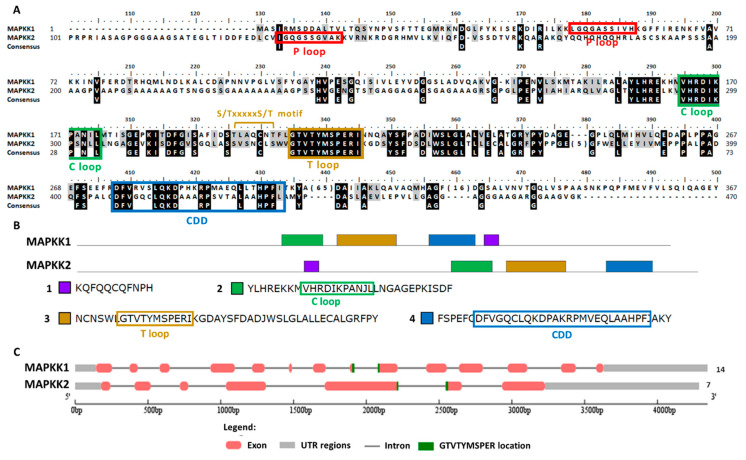
Sequence alignment and motif analysis of MAPKK family. (**A**) Alignment of the two Chlamydomonas MAPKKs identified. The motifs S/TxxxxxS/T, the P, C and T loops and CDD are highlighted. (**B**) Domain analysis in MAPKKs proteins. The two MAPKKs are shown with the consensus sequence identified in MEME (4 domains, listed in order of appearance from the N-terminal). The conserved sequences of the different motif are highlighted in colored rectangles. (**C**) Analysis of the MAPKKs genes sequence with the GSDS program. The distribution of exons and introns of the MAPKKs genes sequences in kilobases (kb) is shown. The 5′ and 3′untranslated regions (UTR), the location of the activation sequence (GTVTTYMSPER) and at the end the number of exons is shown.

The MAPKKs alignment showed the activation domain or the T loop, the C loop, the P loop and the CDD (Figure 6A). In the domain analysis, four domains were observed (Figure 6B and Table S2). In both MAPKKs the activation sequence is located between exons (Figure 6C). In plants the phylogenetic classification of MAPKKs establishes four groups (A–D) [35]. Group B is a very characteristic group since it is MAPKK with a NTF domain [51], which therefore includes Chlamydomonas MAPKK1. Chlamydomonas MAPKK2 presents high identity to Arabidopsis MAPKK6 classified in group A.

### 2.5. Chlamydomonas MAPKK Gene Expression

**Figure 7:**
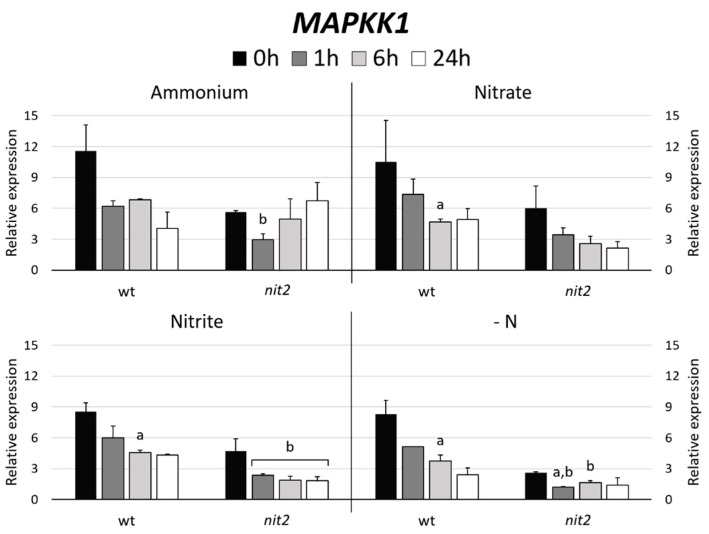
*MAPKK1* gene expression. The gene expression of Chlamydomonas *MAPKK1* was studied in the wt (704) and *nit2* (89.87), other conditions as in Figure 4.

In Arabidopsis, MAPKK5 phosphorylates MAPK6 [41]. Therefore, the gene expression of the Chlamydomonas MAPKK most closely related to Arabidopsis MAPKK5, which turned out to be MAPKK1 (47%) versus MAPKK2 (37%) was studied. The expression of *MAPKK1* in most N sources decreased over the time. However, it is noteworthy that *MAPKK1* expression in *nit2* was always lower than in the wt, especially in -N medium (Figure 7). Similar expression patterns were obtained when the *MAPK3* and *MAPK8* gene expressions were studied (Figure 4). These data indicate a role of *NIT2* controlling the expression of *MAPKK1*, *MAPK3* and *MAPK8*.

### 2.6. Chlamydomonas MAPKKK Family

The MAPKKK group is subdivided into MEKK-, RAF- and ZIK-type MAPKKKs [33]. To search MEKK-type MAPKKKs, the conserved sequence of the activation motif G(T/S)Px(W/Y/F)MAPEV was used [52]. This search generated 11 MEKK-type MAPKKKs. To search RAF-type MAPKKKs, the conserved sequence of the activation motif GTxx(W/Y)MAPE was used [52]. This search generated 94 RAF-type MAPKKKs. To search for ZIK-type MAPKKKs, the conserved sequence of the activation motif GTPEFMAPE(L/V)Y was used [52]. This search generated three ZIK-type MAPKKKs. The vast majority of these putative MAPKKKs genes were not annotated or erroneously annotated in the Phytozome database. Therefore, we have annotated them according to the position they occupy in the different chromosomes. The main characteristics of all identified MAPKKKs are summarized in Table S1.

**Figure 8:**
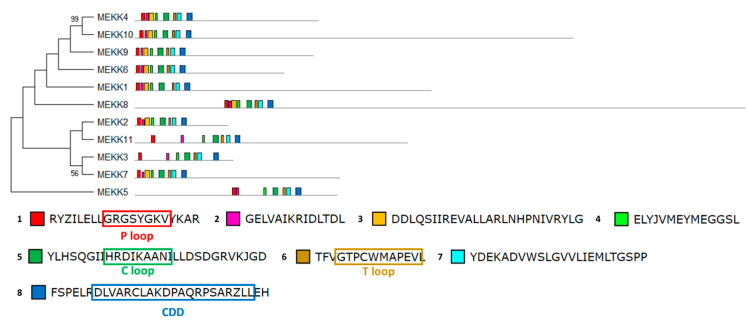
Phylogenetic and motif analysis of MEKK-type MAPKKKs. The MEKK-type MAPKKKs classified by maximum likelihood phylogenetic analysis is shown on the left. On the right, the analysis of their domains identified with the MEME program. Below, the conserved sequence of the identified domains. For other details see Figure 2.

**Figure 9:**
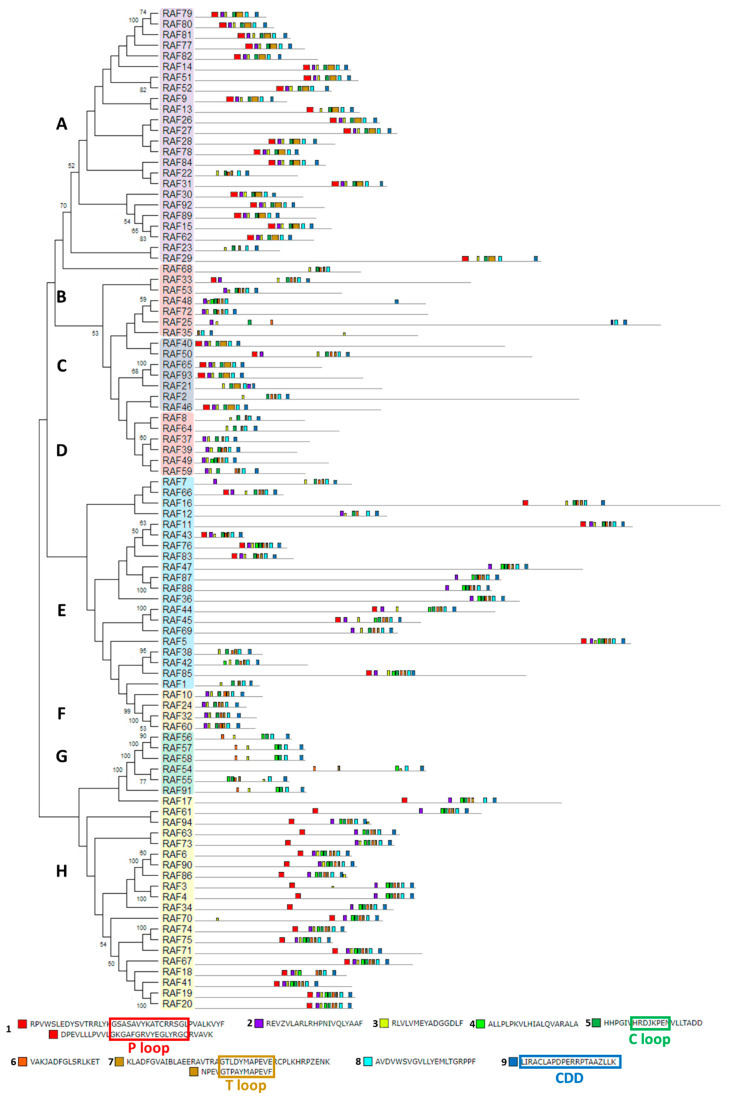
Phylogenetic and motif analysis of RAF-type MAPKKKs. The RAF-type MAPKKKs classified by phylogenetic analysis is shown on the left. Each phylogenetic group has a different background color. On the right, the analysis of their domains identified with the MEME program. Below, the conserved sequence of the identified domains. For other details see Figure 2.

**Figure 10:**
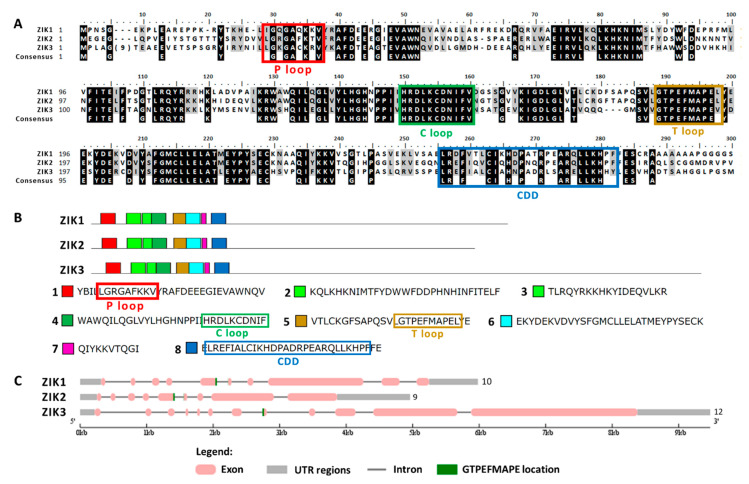
Sequence alignment and motif analysis of ZIK-type MAPKKKs. (**A**) Alignment of the 3 Chlamydomonas ZIK-type MAPKKKs identified. The P, C and T loops and the CDD are highlighted. (**B**) Domain analysis in MAPKKKs type ZIK. The 3 ZIK MAPKKKs are shown with the consensus sequence identified in MEME (the domains are listed in order of appearance from the N-terminal). (**C**) Analysis of the MAPKKKs ZIK gene sequence with the GSDS program. For other details see Figure 6.

The structural study of MEKK-type MAPKKKs identified its main motifs, the P, C and T loop, and the CDD domains (Figure 8 and Table S2). These domains are located mainly at the N-terminal domain, except in MEKK8 and MEKK5. Phylogenetically, the MEKK proteins did not show a high percentage of similarity, probably because of its long C-terminal domains. The phylogenetic classification of RAF-type MAPKKKs generated eight branched groups (A-H) (Figure 9 and Table S2). Group A is characterized by the conserved sequence GTxxYMAPEV in the C loop, the conserved sequence GxxSxVYxAxCxxSGxxVALK in the P loop and a long N-terminal domain. Group C showed the same characteristics as group A, and a long C-terminal domain. In groups B, D and E, the activation site shows a heterogeneous sequence. The phylogenetic differences within these groups were observed at the N- and C-terminal domains. Within Group F, proteins presented a high percentage of similarity and short length. Group G had the conserved sequence GTRHYMAPEV at the activation site. Group H is characterized by the conserved sequence GTxxYMAPE(A/C/L/T) and GxGxxGxV in the activation site and in the P loop, respectively. The structural study of ZIK-type MAPKKKs identified its main motifs, the P, C and T loop, and the CDD domains (Figure 10 and Table S2). In the domain analysis, eight domains were observed. The predicted subcellular location was nuclear for ZIK1 and ZIK3 and ambiguous for ZIK2, the rest of the properties are compiled in Table S1.

Figure S2 shows the distribution of all the MAPK cascade components on the Chlamydomonas chromosomes. Three pairs of MAPKs are located in tandem: MAPK6-MAPK3 on chromosome 12; MAPK9-MAPK10 on chromosome 1 and MAPK16-MAPK17 on chromosome 17. Likewise, eight pairs of RAF are located in tandem. RAF3-RAF4 on chromosome 1, RAF19-RAF20 on chromosome 3, RAF26-RAF27 and RAF28-RAF29 on chromosome 4, RAF44-RAF45 on chromosome 9, RAF51-RAF52 on chromosome 10, RAF74-RAF75 on chromosome 16 and RAF87-RAF88 on chromosome 17. ZIK2 and ZIK3 are located in tandem on chromosome 1. Two clusters of RAF were identified. One cluster of 5 genes in chromosome 11 (RAF54, RAF55, RAF56, RAF57, RAF58), which formed almost the entire phylogenetic group G (except of RAF91); and the other was located in chromosome 16 (RAF79, RAF80, RAF81). In some of the proteins located in tandem the percentage of protein similarity was very high, which suggests gene duplication events (Figure S2).

### 2.7. Gene Expression of MAPKKK Candidates to be Involved in the N Assimilation

**Figure 11:**
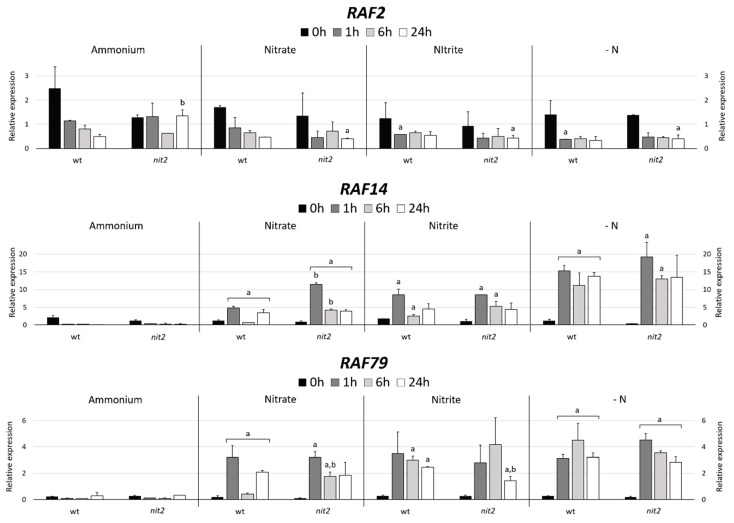
Quantification of *RAF2*, *RAF14* and *RAF79* expression. The expression of MAPKKKs *RAF*2, *RAF14* and *RAF79* were studied in the wt (704) and *nit*2 (89.87), other conditions as in Figure 4.

In order to find MAPKKKs potentially related to N metabolism, two strategies were performed. Firstly, MAPKKKs genes co-expressed with NIT2, the main regulator for nitrate assimilation in Chlamydomonas, were searched using an in-silico tool (Phytozome); and secondly, different transcriptomics data available in Chlamydomonas were analyzed. *RAF2*, the third gene with the highest level of co-expression (Figure S3), was chosen as a MAPKKK candidate related to N assimilation. Since *NIT2* expression is strongly repressed by ammonium [18], the same would be expected for *RAF2* if indeed is co-expressed with *NIT2*. However, *RAF2* showed a high expression in ammonium compared to -N (Figure 11). In both strains, in nitrate, nitrite and –N media the expression of *RAF2* declined after the first hour and then remained constant. However, this gene expression in ammonium decreased in the wt but was maintained in *nit2* mutant. This suggests that RAF2 may have a role in response to ammonium signal.

In a previous transcriptomic study on Chlamydomonas during N deprivation, 118 transcripts were found to be upregulated at the early-stage response, from 0 to 18 min [53]. These transcripts were associated with signaling, modulation of growth or stress response; interestingly two of them were *RAF14* and *RAF79*. Therefore, we decided to analyze the expression of these two genes. The expression of these genes was highly repressed by ammonium in both strains (Figure 11). However, the expression of *RAF14* and *RAF79* in nitrate, nitrite and especially in -N was notably increased in both strains. These data indicate that *RAF14* and *RAF79* are strongly repressed by ammonium and that their expressions were independent of *NIT2*. Therefore, RAF14 and RAF79 may be involved in sensing the absence of ammonium and participate in the regulation of N metabolism.

## 3. Discussion

MAPK cascades are the final transmitters of stimuli from outside into the cells. The main objective of this work was to discover if the MAPK cascade has any relationship with N metabolism using the alga Chlamydomonas as a model organism. For this purpose, each component of the different MAPK families in Chlamydomonas were identified and characterized structurally and phylogenetically, and their possible role in the regulation of N assimilation of selected MAPKs members of each family studied. In the MAPK cascade analysis 17 MAPKs, two MAPKKs, 108 MAPKKKs (11 MEKK-, 94 RAF- and three ZIK-types) were identified. The number of MAPKs identified in Chlamydomonas was similar to other model organisms such as Arabidopsis (20) or rice (17) [36]. Several studies have identified MAPKs in algae [35,42,43,47]. However, the number of MAPKs identified in those works were low (5–6) compared to our study, because only MAPKs with TEY or TDY in its activation motif were considered. However, our data are consistent with a study in Chlamydomonas about the function of iron deficiency that identified 16 MAPKs [44], that like us also considered as real MAPKs those with TTY, TSY and TPY in the activation motif. Consequently, this work expands the number of identified MAPKs and establishes new TxY sequence motifs, TTY, TSY and TPY present in the alga, in addition to the commonly described TEY and TDY. Only two Chlamydomonas MAPKKs were identified in contrast to the 10 and seven MAPKKs previously identified in Arabidopsis and rice, respectively. However, in other algae, a similar number of putative MAPKK was found: two in *Volvox carteri*, two in *Micromonas sp. RCC299*, one in *Ostreococcustauri* and one in *Ostreococcus lucimarinus* [45].

Based on a phylogenetic analysis, we have divided Chlamydomonas MAPKs into three groups. In Arabidopsis, group A MAPK, such as MAPK3 and MAPK6, have a function related to the development or response to stresses. Group B, such as MAPK4, have been related to the defense response to pathogens. Group C do not have a clear function assigned yet. In addition, it has been shown that MAPKs of the A–C groups participate in canonical MAPK cascade pathways. Group D MAPKs are characterized by having a very poorly conserved CDD and a long and variable C-terminal domain [33]. In contrast to Group A–C, it has been suggested that group D may be involved in additional functions [33]. Table S1 collect the predicted subcellular location of every MAPK component, however the MAPK location is not necessarily indicated only in the primary sequences and may depend on MAPK interaction with regulators, scaffold proteins, cytoskeletal proteins or transcription factor [34], that alter the predicted in silico location.

In Chlamydomonas some MAPKs have been related to the cell cycle regulation, in this sense, the expression profiles of *MAPK4*, *MAPK5* and to a lesser extent *MAPK2*, *MAPK6* and *MAPK8* peak at the beginning of alternating synthesis and mitosis phase (S/M phase) followed by a drop in expression [43]. Results that are in agreement with RNA-seq transcriptomics where more pronounced expression peak at S/M phase were detected for *MAPK6* and *MAPK8* [54]. The Pharmacological inhibition of MAPK cascade by PD98059 have been shown in Chlamydomonas [46]. Therefore, the implication of algal MAPKs in the cell cycle regulation could be subject to further studies. In contrast, in three different algal species *Chlamydomonas reinhardtii*, *Chlorella vulgaris*, and *Haematococcus pluvialis*, MAPK cascade are negatively implicated in both lipid and carotenoid biosynthesis [55].

Several findings suggest that the MAPK cascade may have an important role in the N assimilation and signaling. For instance, an in vivo phosphoproteomic study during N starvation in Chlamydomonas reveals a high increase in the presence of phosphorylated SP motif, the MAPK phosphorylation motif, which suggest a central role of MAPK cascades in the acclimatization to N availability [56]. In Arabidopsis, the MAPK cascade is involved in regulating the NR to control NO production. In response to H_2_O_2,_ MAPK6 phosphorylates NR, what causes an increase of NO production [41]. In response to nitrate, the Arabidopsis transcription factor NLP7, the master regulator of early nitrate signaling in roots, binds to the promoter regions of five MAPKKKs MEKK-type (MAPKKK13, 14, 17, 18 and 19) [57]. Arabidopsis MAPKKK8 (also named MEKK1) is involved in the glutamate signal to modulate root architecture [58]. This protein has also been proposed to act as an activator of MAPK6 [59]. Since MAPK6 phosphorylates NR [41], glutamate could have a feedback role controlling the NR activity.

In a systematic search in Arabidopsis, 570 potential MAPKs phosphorylation targets have been identified [60], some of them involved in regulating the N metabolism, like NR2 (phosphorylated by MAPK7) and CIPK23 (phosphorylated by MAPK16). CIPK23 is a kinase that regulates the nitrate transceptor NRT1.1, which is a essential for both nitrate transport and signaling [61]. Interestingly, NR2 was found to be phosphorylated by MAPK7 in that study. Therefore, Arabidopsis N assimilation could be regulated by more than one MAPK (MAPK6 and MAPK7). In this sense, the enzymatic activity of some MAPK has been found to be redundant. In Arabidopsis, MAPK6 and MAPK3 are redundantly involved in various signaling cascades related to abiotic stresses, inmate immunity, ethylene response and stomatal development [62]. That may also be the case for NR regulation, with more than one MAPK involved in its phosphorylation. This might be the reason why when we analyzed the Chlamydomonas *mapk8* mutants only slight effects were observed in the growth on the different N sources. In this sense, if in Chlamydomonas there is another MAPK redundant to MAPK8, only by creating the double mutant, a significant effect could be observed.

The four Chlamydomonas MAPKs and MAPKK1 analyzed decreased slightly its gene expression over time in all the N sources studied. Interestingly, the expression of *MAPKK1*, *MAPK8* and *MAPK3* seem to be somehow related to nitrate assimilation, since they needed *NIT2* to sense the absence of N. These data indicate that MAPK and MAPKK have a slight transcriptional regulation by N. This suggests that the main regulation mechanism of MAPK and MAPKK is post-translational, consistent with the MAPK cascade, in which MAPK is activated by MAPKK phosphorylation and MAPKK is activated by MAPKKK phosphorylation. These phosphorylations are reverted by phosphatases [63], establishing a clear post-translational regulation of MAPKs and MAPKKs, triggered by an initial activation of the MAPKKK protein. Even more, a single MAPK can be regulated by more than a single MAPKKK/MAPKK pair [64]. Therefore, the best candidates of the cascade to be transcriptionally regulated are MAPKKK, which are the first to respond to stimuli.

In the genome of all organisms MAPKKKs represent the largest group of MAPK family. In Chlamydomonas, we have identified 108 MAPKKKs. This number is comparable to that identified in other organisms, 75 in rice, 74 in tomato and 89 in Arabidopsis [36]. Therefore, the large number of MAPKKKs identified in Chlamydomonas contrasts with the low number of MAPKKs, so it seems very unlikely that all MAPKKKs identified participate in canonical MAPK routes. In plants, the MAPKKK family of proteins has not been well characterized yet, especially the RAF-type MAPKKKs. In addition, it has not yet been confirmed that any RAF- or ZIK-type are capable of phosphorylating MAPKK, so it is questioned whether they actually participate in canonical MAPK pathways or fulfill additional functions despite retaining the characteristic MAPKKK domains [40]. Studying the regulation of the genetic expression of 3 RAF-type MAPKKKs candidates to be involved in the N assimilation was observed that *RAF14* and *RAF79* are strongly repressed by ammonium, independently of NIT2. Therefore, *RAF14* and *RAF79* could be playing a role as ammonium sensors. In fact, the previously mentioned Chlamydomonas phosphoproteomic study also revealed that RAF14 phosphorylation level, increased by N starvation, is decreased during N replenishment [56]. More experiments will be needed to test this hypothesis, but the results are promising. If confirmed, RAF14 and/or RAF79 would be the first MAPKKK proteins involved in N signaling/assimilation in algae. In the Chlamydomonas genome, *RAF79* was found in a cluster with *RAF80* and *RAF81* in chromosome 16. In this sense, *RAF80* and *RAF81* may have a similar expression and function.

To date, only some post-translational regulation mechanisms of NR have been described in detail. In Arabidopsis, NR inhibition by phosphorylation of serine 534 in the hinge 1 by 14-3-3 protein binding has been demonstrated [65]. Additionally, it has been shown that MAPK6 phosphorylates the serine 627 of NR located in the hinge 2. Since this serine 627 is also conserved in NR of other plants, this post-translational regulation of NR may be controlling the production of NO [66]. In Chlamydomonas, NR together with its accessory proteins NOFNiR and THB1 regulate the NO level; however, the mechanisms that govern the interaction of these proteins in the regulation of NO levels are unknown. 

**Figure 12:**
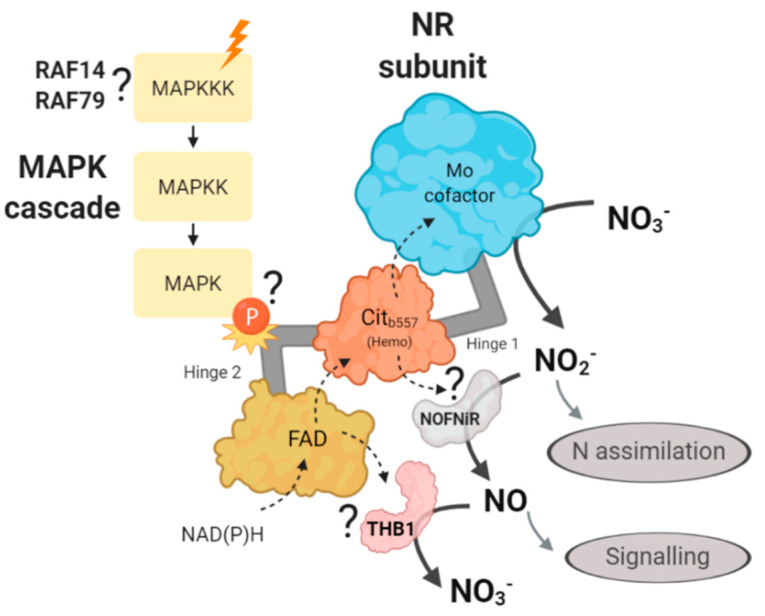
Illustration of the hypothetical mechanism proposed about the role of the MAPK cascade in the regulation of nitrate reductase. The different N sources (orange ray) would activate a MAPK signaling cascade that would ultimately cause the phosphorylation of NR, probably in the hinge region 2. This would activate/inhibit the binding of NOFNiR or THB1, which would control the reduction of nitrite to NO, and would act as a signaling molecule. With our data we propose RAF14 and RAF79 as candidates to sense the N signal. The question mark indicates that such interactions have not been experimentally demonstrated yet. The dotted arrows represent the movement of electrons and the solid arrows the substrate and product of each protein.

We propose that NR hinge 2 could be involved in the interaction with THB1 or NOFNiR. Therefore, in the following hypothetical mechanism, we propose that a MAPK cascade through the NR phosphorylation may be affecting the binding of NR with NOFNiR or with THB1 (Figure 12). Thus, a MAPK cascade may be controlling the production of NO and therefore, the regulation of N metabolism in Chlamydomonas. Based on the data generated in this study, we propose that the MAPKKKs involved in this regulation are RAF14 and RAF79.

## 4. Materials and Methods

### 4.1. Chlamydomonas Strains and Growth Conditions

The Chlamydomonas strains used here were 704, 21gr (CC-1690) and 6145c (CC-1691) wild types (wt) for N assimilation, and 89.87 a *NIT2* mutant [18]. The Chlamydomonas *mapk8* mutant LMJ.RY0402.172590 and its parental strain CC-5325 (*nia1nit2*) were obtained from the Chlamydomonas Resource Center (https://www.chlamycollection.org/). Chlamydomonas cells were grown at 25°C under continuous light in Tris-Acetate-Phosphate (TAP) ammonium medium as previously described [1]. At the mid-exponential phase of growth, cells were collected by centrifugation (4000× *g*, 5 min), washed twice with TAP without N and transferred to medium containing the indicated N source, either ammonium (NH_4_Cl), nitrate (KNO_3_), nitrite (KNO_2_) or without N. After induction, cells were collected by centrifugation and processed immediately for enzyme assays, RNA extraction, or analysis [31].

### 4.2. Chlamydomonas Genetic Cross

The genetic cross between wt 21gr and *mapk8* mutant was performed by the random spore plating method [67]. The cross was performed to change the *nia1nit2* mutant background to a wt background. One hundred randomly selected colonies were plated on TAP agar (1.6%) plates containing ammonium (8 mM) as sole N source with or without paromomycin (25 μg·mL^−1^) for segregants analysis. Two *mapk8* mutants, *mapk8-1* and *mapk8-2*, and wt for N assimilation were obtained.

### 4.3. Nitrate Reductase Activity

The NADPH dependent NR activity was measured with 100 µL extracts, prepared form 10 mL culture, according to reported methods [31].

### 4.4. Quantification of Chlamydomonas Growth

The cells were grown under contiguous light with the selected N sources and at the indicated times the chlorophyll content was measured [68].

### 4.5. Quantification of Gene Expression by RT-qPCR

Total RNA was purified as in previously reported protocols [69] and cDNA was synthesized using iScript Select cDNA Synthesis Kit (BioRad, Hercules, CA, USA). Primers for quantification of gene expression (Table S3) were designed using the quantPrime tool [70] and analyzed with the Primerselect program. For the housekeeping gene specific primers (Ubiquitine ligase, Ubiupper and Ubilower) were used [71]. The RT-qPCR was performed on the LightCycler Instrument (iCycler iQ5 real-time PCR detection system; Bio-Rad) using SsoFast EvaGreen Supermix (Bio-Rad). Mean comparisons were made by the Student’s t-test taking into consideration ammonium as the reference medium and wt as the reference strain. Then, statistical significance (*p* < 0.05) is highlighted between nitrate, nitrite or without N and ammonium medium of the same time and strain sample with a; and between *nit2* mutant and wt of the same time and medium sample with b.

### 4.6. MAPK Cascade Identification 

First, to identify MAPK cascade proteins in the *Chlamydomonas reinhardtii v5.6* genomewe used BLAST (https://blast.ncbi.nlm.nih.gov/Blast.cgi) with conserved sequence of the activation domains in the Phytozome (https://phytozome-next.jgi.doe.gov/) and NCBI (National Center for Biotechnology Information) (www.ncbi.nlm.nih.gov) databases. The results from both databases were trimmed. Furthermore, Hidden Markov Model (HMM) profiles were built based on the Arabidopsis MAPK cascade with *hmmbuild* tool of the HMMER 3.0 (http://hmmer.org/download.html). The 20 Arabidopsis MAPK protein sequences were obtained from TAIR10 (https://www.arabidopsis.org/) and were aligned with ClustalW (https://www.genome.jp/tools-bin/clustalw). The alignment was used to built a HMM profile for the MAPK search on Chlamydomonas. The same was done for the 10 MAPKKs, 21 MEKKs, 48 RAFs and 11 ZIKs of Arabidopsis obtaining five HMM profiles. Each HMM profile was used to search on a local protein database (*Chlamydomonas reinhardtii v5.6*). Proteins identified from BLAST were confirmed with HMMER results. Then, the presence of the protein kinase domain was confirmed in all the obtained putative MAPK cascade with hmmscan online tool (https://www.ebi.ac.uk/Tools/hmmer/search/hmmscan) and Pfam database. Moreover, the theoretical pI and molecular mass were determined with Protparam (https://web.expasy.org/protparam/) and subcellular location with Cello2go (http://cello.life.nctu.edu.tw/cello2go/). Chromosomal location was obtained from phytozome and is shown in Figure S2 and Table S1.

### 4.7. Phylogenetic and Domain Analysis

Evolutionary trees were constructed using the Maximum Likelihood method and default settings in MEGA7 [72] and the JTT matrix model [73]. Alignments were performed using the ClustalW method, and the evolutionary distances were computed using the Poisson correction model [74]. The bootstrap consensus tree was inferred from 1000 replicates [75]. The construction of the MAPK, MEKK and RAF MAPKKKs trees involved 17, 11 and 94 sequences, respectively. MEME (Multiple Expectation maximization for Motif Elicitation) (http://meme-suite.org/) was used for the detection of domains conserved in protein sequences and GSDS (Gene Structure Display Server) (http://gsds.cbi.pku.edu.cn/) for the detection of exons and introns in nucleotide sequences. Furthermore, phylogenetic analysis in Figure S1 involved 66 sequences from Chlamydomonas, Arabidopsis, rice (Phytozome), yeast and human (NCBI).

### 4.8. Additional Bioinformatics Tools

Bioedit software was also used for the alignment of protein sequences. DNAStar (Lasergene) program package (https://www.dnastar.com/) includes: Seq Builder, analysis of restriction enzyme target sites; SeqMan, management and analysis of DNA sequencing results; PrimerSelect, primers design and Biorender (https://biorender.com/) for image design.

## Supplementary Materials

Supplementary materials can be found at https://www.mdpi.com/1422-0067/21/10/3417/s1. Figure S1. Phylogenetic analysis of MAPK from Chlamydomonas, Arabidopsis, rice, yeast and human. Figure S2. Chromosomal locations of MAPK cascade genes in Chlamydomonas. Tandem proteins are framed and similarity percentage is shown on the right. Figure S3. Co-expression of *NIT2* and *RAF2* (Cre01.g003800). Table S1. Summary of the main characteristics of all the MAPK cascade members identified. Table S2. Main consensus motif in the MAPK cascade of Chlamydomonas. Table S3. Primers lists.
