# Supplementary material for: Identification of the MAPK Cascade and its Relationship with Nitrogen Metabolism in the Green Alga Chlamydomonas reinhardtii
Source: Int J Mol Sci. 2020 May 12;21(10):3417. doi: 10.3390/ijms21103417 (PMC7279229; doi:10.3390/ijms21103417)
Supplement: Supplementary file 1 [file ijms-21-03417-s001.pdf]

Table S1. Summary of the main characteristics of all the MAPK cascade members identified. 17 MAPKs, 2 MAPKKs, 11MEKK-like, 94 RAF-like and 3 ZIK-like MAPKKKs were identified. The name, protein ID (NCBI) and gene (Phytozome), chromosomal location, gene length, ORF (open reading frame) length, CDS (coding sequences) length, number of exons, size are shown from left to right of the protein, its molecular weight and the isoelectric point (pI) determined with protparam (ExPASy), the location of the kinase domain in the amino acid sequence and, finally, the subcellular location, predicted from Cello2go.M (plasma membrane), E (extracellular), Cl (chloroplastic), N (nuclear), C (cytoplasmic), Mi (mitochondrial).

**Family (subfamily)**	**Name**	**Protein ID (NCBI)**	**Gen ID (phytozome)**	**Chromosomal location**	**Gene length (bp)**	**ORF length (bp)**	**CDS length (bp)**	**Number of exons**	**Protein size (aa)**	**Molecular weight**	**pI**	**Kinase domain location (aa)**	**Subcellular location**
MAPK	MAPK1	PNW84924	Cre03.g164250	3041386	9374	7143	6285	11	2094	209,92	4,53	1496-1797	N
	MAPK2	PNW80276	Cre08.g385050	4906426	6897	4200	2223	12	740	79,02	8,61	151-488	N
	MAPK3	XP_001690852	Cre12.g509000	2119590	2446	1110	1062	8	383	43,03	6,65	45-381	C/ Mi/ N
	MAPK4	PNW71073	Cre17.g745447	6855222	7528	5201	2298	9	765	81,21	7,2	60-400	N
	MAPK5	XP_001699095	Cre13.g607300	5094976	4412	2454	1302	11	433	48,54	8,88	6-343	C/ Mi/ N
	MAPK6	XP_001690850	Cre12.g508900	2129460	3866	2690	1128	8	375	42,52	7,66	37-369	C/ Mi
	MAPK7	PNW71601	Cre16.g661100	2519399	4622	2587	1758	7	585	63,32	9,7	4-283	N
	MAPK8	XP_001700291	Cre01.g010000	1838122	4166	2025	1170	10	389	43,86	5,68	52-389	C/ N
	MAPK9	PNW88973	Cre01.g052800	7342585	9116	5569	3462	12	1163	117,97	9,91	2-269	N
	MAPK10	PNW88974	Cre01.g052850	7356808	10268	7895	5199	7	1732	168,69	9,5	2-252	N
	MAPK11	PNW87149	Cre02.g111014	5820048	7742	5461	4212	9	1403	142,51	9,15	21-281	N
	MAPK12	ALE15285	Cre03.g169500	3709775	6332	4661	3321	7	1106	114,21	9,49	2-289	N
	MAPK13	PNW85915	Cre03.g200200	8184080	8704	7281	5301	6	1766	176,78	9,36	2-289	N
	MAPK14	PNW77338	Cre10.g432250	1948371	8496	6947	4752	6	1583	160,55	8,31	2-350	N

	MAPK15	PNW75720	Cre12.g538300	6473633	5596	3795	1923	10	640	68,44	9,11	20-311	N
	MAPK16	PNW70187	Cre17.g709500	1790415	8269	5742	1078	10	1078	107,47	7,93	9-317	N
	MAPK17	PNW70192	Cre17.g709750	1821974	9617	8208	4956	6	1651	165,08	8,56	1-264	N
MAPKK	MAPKK1	XP_001696437	Cre06.g249150	51943	4344	2216	1359	7	452	50,28	6,87	42-305	C/ Mi
	MAPKK2	PNW73553	Cre13.g563733	250093	4286	2657	1422	14	473	47,34	8,16	266-438	N
MAPKKK (MEKK)	MEKK1	PNW88153	Cre01.g016570	2796247	12436	5895	4575	18	1524	150,2	6,61	10-289	N
	MEKK2	PNW86163	Cre02.g075350	311027	4953	2881	1446	11	481	51,47	9,95	15-272	N
	MEKK3	PNW86180	Cre02.g076000	398374	6436	2575	1524	11	507	53,87	6,18	167-435	N
	MEKK4	PNW84939	Cre03.g164900	3111653	8583	3995	2838	15	945	97,18	5,84	35-301	M/ Cl
	MEKK5	PNW80411	Cre07.g316650	578795	12676	5707	3129	24	1042	111,56	8,78	509-857	Mi/ Cl
	MEKK6	PNW80428	Cre07.g317300	666135	6816	3574	2313	14	770	77,8	6,48	10-265	M/ N
	MEKK7	PNW78821	Cre09.g391245	3739567	6446	4603	3171	9	1056	105,22	7,26	21-271	M/ N
	MEKK8	PNW79200	Cre09.g405900	6180085	13190	11042	9672	10	3223	319,55	5,93	466-712	N
	MEKK9	PNW76185	Cre12.g544400	8905993	6640	4404	2760	11	919	89,24	6,49	7-285	N
	MEKK10	PNW76037	Cre12.g550300	8249636	10362	7237	6756	12	2251	222,05	8,62	28-296	N
	MEKK11	PNW74390	Cre13.g606600	4985136	5702	4858	4206	7	1401	142,06	8,75	351-548	N
MAPKKK (RAF)	RAF1	PNW87799	Cre01.g002550	445086	6553	3595	1182	8	393	42,89	7,57	83-354	C/ Mi
	RAF2	PNW87836	Cre01.g003800	726049	10442	8169	6942	8	2313	223,37	8,82	412-569	N
	RAF3	PNW88041	Cre01.g011800	2154460	8416	5100	3993	16	1330	135,57	6,35	1082-1321	E/ N
	RAF4	PNW88042	Cre01.g011850	2162976	9882	5927	3984	16	1327	135,01	6,34	1081-1320	M/ N

RAF5	PNW88330	Cre01.g024750	3819867	12586	9189	7890	24	2629	271,96	5,69	2341-2595	E/ N
RAF6	PNW88608	Cre01.g036900	5276708	9135	5312	2844	14	947	98,96	5,82	630-934	E/ M/ N
RAF7	PNW88718	Cre01.g041550	5816648	6773	5623	2856	5	951	91,94	6,42	54-150 669-859	N
RAF8	PNW88814	Cre01.g045600	6392567	7616	2513	1998	17	665	70,85	6,54	133-397	Mi
RAF9	PNW86144	Cre02.g074850	250404	5926	4370	1671	7	556	58,52	8,89	200-479	N
RAF10	XP_001701841	Cre02.g075900	379966	5145	2423	1227	11	408	45,6	6,88	19-301	C/ Mi/ N
RAF11	PNW86537	Cre02.g090700	2322421	12416	8235	7920	16	2639	255,14	7,28	2336-2594	N
RAF12	PNW86746	Cre02.g095129	3546432	9678	5007	3486	13	1161	118,3	8,86	842-1113	N
RAF13	PNW87185	Cre02.g112333	6011184	6162	5043	2982	6	993	101,44	9,08	681-961	M/ N
RAF14	PNW87190	Cre02.g112500	6026336	6172	4888	2820	7	939	96,27	9,36	659-925	N
RAF15	PNW87213	Cre02.g113600	6154135	5741	4040	2478	10	825	89,91	8,95	450-717	N
RAF16	PNW84391	Cre03.g144011	112790	19766	13774	12096	22	4031	391,21	9,09	1986-2048; 2238-2475	N
RAF17	PNW84416	Cre03.g144464	254796	10582	8392	6639	11	2212	222,84	7,23	1516-1817	N
RAF18	PNW84612	Cre03.g151300	1431578	8446	4249	2745	15	914	93,05	7,76	537-850	M
RAF19	PNW84795	Cre03.g159016	2402996	9645	4948	2907	20	968	100,98	6,41	690-939	M/ N
RAF20	PNW84801	Cre03.g159150	2418801	8690	4836	2859	22	952	99,78	6,48	687-945	M/ N
RAF21	PNW85039	Cre03.g169100	3671907	5977	4973	3393	6	1130	114,53	9,71	151-368	N
RAF22	PNW85396	Cre03.g184450	5367591	4648	3613	1866	8	621	67,23	6,3	63-331	N
RAF23	XP_001693326	Cre03.g205950	7421375	5925	4356	1539	6	512	54,87	8,99	166-433	N

RAF24	XP_001699403	Cre03.g209505	8986322	4924	2893	846	5	312	35,81	7,64	3-268	C/ Mi/ N
RAF25	PNW84074	Cre04.g220000	2201172	20466	10261	8424	24	2807	261,46	10,73	46-144; 295-338; 2505-2603	N
RAF26	PNW84283	Cre04.g228208	3529518	6586	4718	3351	10	1116	113,32	8,08	821-1102	N
RAF27	PNW84286	Cre04.g228300	3540468	8426	6572	3660	8	1219	123,04	9,31	906-1189	N
RAF28	PNW84299	Cre04.g228800	3649997	8388	5881	2538	8	845	84,13	9,03	453-740	M/ N
RAF29	PNW84300	Cre04.g228850	3658847	9703	8103	6261	7	2086	208,72	7,04	1626-1845; 2031-2069	N
RAF30	PNW83575	Cre05.g235450	2454358	5939	3101	1965	12	654	69,2	5,45	225-493	N
RAF31	PNW82378	Cre06.g278265	4349283	6243	4464	3471	10	1156	118,73	8,4	833-1138	N
RAF32	XP_001701605	Cre06.g292700	6447570	4589	2294	1119	10	372	41,25	6,64	14-278	C/ Mi/ N
RAF33	PNW82768	Cre06.g293300	6524617	10730	8168	4986	10	1661	164,06	8,97	110-157; 489-693	N
RAF34	PNW80504	Cre07.g320050	1101146	8133	5674	3591	14	1196	121,8	5,84	928-1155	M
RAF35	PNW80614	Cre07.g324932	1601084	7632	6506	4035	4	1344	129,69	6,67	20-112	N
RAF36	PNW80665	Cre07.g325739	1880192	11746	6909	5886	22	1961	196,49	5,38	1612-1872	M/ N
RAF37	XP_001690456	Cre07.g328850	2419195	7439	3406	2082	16	693	72,23	5,79	4-260	C/ N
RAF38	PNW81253	Cre07.g348450	5176903	4142	3394	1242	5	413	45,43	7,99	70-349	Mi
RAF39	XP_001692604	Cre07.g351150	5523427	3936	2711	1857	7	618	66,62	9,34	17-278	N
RAF40	PNW79609	Cre08.g359900	623303	11281	8521	5607	10	1868	183,11	8,26	11-278	N
RAF41	PNW79945	Cre08.g373300	2692638	5823	3787	2844	16	947	98,04	8,77	488-744	M/ N
RAF42	PNW80223	Cre08.g382800	4548983	8175	3290	2061	14	686	75,76	5,78	131-396	C/ Mi/ N

RAF43	PNW80273	Cre08.g384900	4886273	3825	1774	903	8	300	33,48	6,76	54-293	C/ Mi/ N
RAF44	PNW78786	Cre09.g390097	3575016	10188	6634	5439	23	1812	186,47	6,21	1076-1139;<br>1410-1577	E/ M/ N
RAF45	PNW78787	Cre09.g390134	3585533	8831	5484	4098	20	1365	144,97	6,17	861-1251	M
RAF46	PNW79090	Cre09.g400330	5483547	8491	5366	3372	12	1123	113,64	9,33	58-325	N
RAF47	PNW78274	Cre09.g404050	513690	14532	8762	7017	27	2338	231,73	6,29	1722-2013	N
RAF48	PNW79373	Cre09.g412750	7279600	12979	5410	4170	25	1389	139,96	8,41	3-225	N
RAF49	XP_001690509	Cre10.g443300	3275379	5032	3184	2439	12	812	88	8,43	3-260	N
RAF50	PNW77652	Cre10.g445700	3645177	9172	7920	6096	9	2031	204,73	7,01	357-413;<br>739-934	N
RAF51	PNW78079	Cre10.g463500	6084346	7572	5980	2958	9	985	99,79	8,73	666-943	N
RAF52	PNW78080	Cre10.g463550	6093116	6642	5364	2475	7	824	84,63	9,02	502-809	M/ N
RAF53	PNW78096	Cre10.g464100	6170561	4585	3645	2664	7	887	92,88	8,11	121-384	N
RAF54	XP_001701400	Cre11.g467584	419312	4675	3619	2082	5	1393	151,11	5,31	684-963;<br>1099-1383	M/ C
RAF55	XP_001698779	Cre11.g467585	424042	4563	2829	1512	4	570	60,32	6,07	197-368;<br>389-560	M/ Cl
RAF56	PNW76439	Cre11.g467586	430502	4460	3744	585	4	585	60,89	4,95	404-575	M/ N
RAF57	PNW76441	Cre11.g467588	439960	6112	5049	2001	5	666	69,46	5,1	485-656	M/ Cl/ N
RAF58	PNW76442	Cre11.g467589	446121	6091	5035	2001	5	666	69,45	5,06	485-656	M/ Cl/ N
RAF59	PNW76806	Cre11.g477000	2718724	8114	3390	2013	11	670	69,64	7,83	17-392	N
RAF60	PNW76909	Cre11.g481000	3434420	5331	2303	1098	9	365	41,1	8,25	13-277	C/ Mi/ N
RAF61	PNW74603	Cre12.g490800	1081687	11319	7352	5181	17	1726	169,37	6,26	1346-1649	N

RAF62	XP_001702346	Cre12.g498650	3229809	5607	4590	2157	4	718	77,03	9,16	323-589	N
RAF63	PNW74924	Cre12.g504250	2653553	9212	6101	3704	15	1237	127,41	6,12	644-667; 948-1214	N
RAF64	PNW75452	Cre12.g527000	5121746	4876	3880	2613	6	870	92,12	6,78	109-380	Cl/ N
RAF65	XP_001696965	Cre12.g529550	5352264	4814	3038	2310	13	769	80,25	9,68	36-303	N
RAF66	PNW73628	Cre13.g566450	699683	6736	3794	1620	11	539	57,48	6,9	186-499	N
RAF67	PNW73650	Cre13.g567200	818679	12619	6697	3939	22	1312	134,71	6,4	910-1169	N
RAF68	PNW73945	Cre13.g579200	2333838	7849	4692	3006	8	1001	98,77	9	683-936	N
RAF69	PNW74069	Cre13.g584100	2975154	9040	5866	3675	13	1224	125,37	6,87	837-1200	E/ N
RAF70	PNW74305	Cre13.g603600	4474934	10146	5379	3399	17	1132	113,4	6,21	822-1110	N
RAF71	PNW74332	Cre13.g604350	4667795	10390	5485	4107	18	1368	133,91	6,92	841-1127	N
RAF72	PNW72926	Cre14.g612000	600655	10262	6555	4221	23	1406	149,32	5,63	3-256	M/ N
RAF73	PNW73252	Cre14.g625050	2479381	9062	5512	3615	18	1204	121,39	7,99	954-1188	M/ N
RAF74	PNW71449	Cre16.g654850	1717261	8561	4357	2748	20	915	95,56	5,82	599-901	E/ M
RAF75	PNW71450	Cre16.g654900	1725871	8756	4464	2496	21	831	87,74	5,55	521-819	M
RAF76	PNW71561	Cre16.g659400	2313335	5179	3264	1677	10	558	61,32	6,02	283-526	C/ Mi
RAF77	PNW72330	Cre16.g672602	6541417	5762	4482	1989	9	662	69,55	9,27	314-586	M/ Cl/ N
RAF78	PNW72306	Cre16.g673600	6415157	7039	5588	1899	8	632	64,51	8,16	364-628	M/ N
RAF79	PNW72379	Cre16.g674065	6774858	3855	3338	1299	4	432	45,72	8,44	109-389	Mi/ Cl
RAF80	PNW72380	Cre16.g674179	6781133	3895	2992	1440	5	479	50,61	7,18	157-437	Cl
RAF81	PNW72381	Cre16.g674291	6785082	5246	4310	1740	6	579	60,35	9,63	268-561	N

	RAF82	PNW72402	Cre16.g676309	6869955	7013	5206	2232	8	743	76,5	6,91	244-527	N
	RAF83	PNW72071	Cre16.g683650	5136056	5002	2505	1797	11	598	62,44	6,05	237-498	Mi
	RAF84	PNW72507	Cre16.g686734	7453925	7009	4965	2367	6	788	81,05	9,54	454-751	N
	RAF85	XP_001691957	Cre16.g687800	4597497	7923	5473	4071	16	2001	209,62	5,7	1046-1330	M/ N
	RAF86	PNW71175	Cre16.g694550	230523	7529	5431	2772	12	923	96,76	7,05	678-896	M
	RAF87	PNW69889	Cre17.g696900	93230	10646	6319	5547	26	1848	187,52	5,73	1563-1835	E/ N
	RAF88	PNW69890	Cre17.g696950	104087	11019	6783	5382	25	1793	182,57	6,03	1508-1780	E/ M/ N
	RAF89	PNW69969	Cre17.g700133	605270	6517	3652	2202	9	733	77,23	9,15	246-512	N
	RAF90	PNW70274	Cre17.g713025	2173220	7466	3682	2937	15	978	101,56	6,15	732-963	M/ N
	RAF91	XP_001700457	Cre17.g713750	2278537	4266	3298	2022	5	673	70,07	5,15	492-663	M/ Cl/ N
	RAF92	PNW70426	Cre17.g719450	2867092	8488	4482	2343	12	780	82,23	9,25	341-609	N
	RAF93	PNW70838	Cre17.g735550	5178500	5900	4698	3045	7	1014	103,55	6,61	29-297	M/ N
	RAF94	PNW70853	Cre17.g736250	5293730	11252	4870	3195	14	1064	107,68	5,49	820-1037	M/ N
MAPKKK (ZIK)	ZIK1	XP_001690141	Cre01.g040150	5637823	4961	3742	2391	10	864	96,86	5,7	17-278	N, M
	ZIK2	PNW88681	Cre01.g040200	5644051	5981	3646	2595	9	796	83,02	5,37	19-279	N
	ZIK3	PNW69970	Cre17.g700166	612184	9473	6107	4794	12	1597	160,43	6,17	27-285	N

Table S2. Main consensus motif in the MAPK cascades of Chlamydomonas. Four motives are shown: loop P, C, T and the CDD domain. A consensus sequence and a conserved sequence are generated from the four MAPK groups, except for the CDD, which conserved sequence is specific to the established MAPK group. In brackets, is shown the number of proteins included in each group and showing that particular consensus sequence.

	P loop	C loop	T loop	CDD
MAPK C (3)	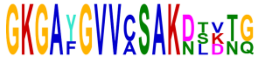	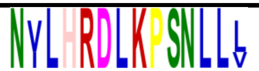	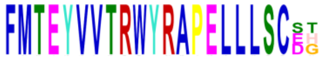	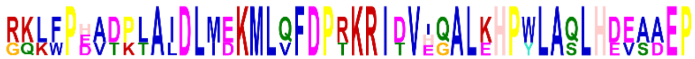
MAPK D (2)	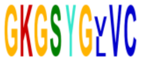	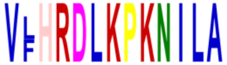	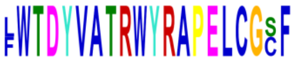	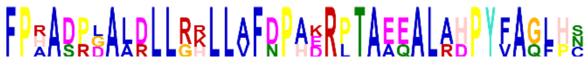
MAPK E (10)	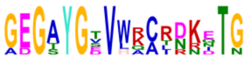	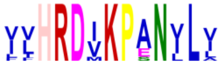	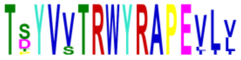	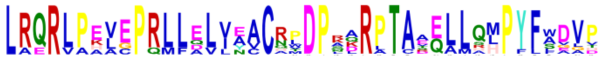
MAPK CONSENSUS	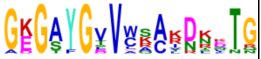	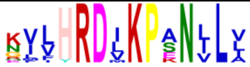	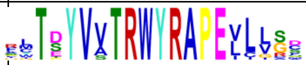	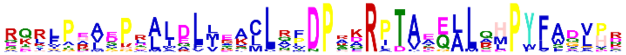
MAPK CONSERVED	-GxGxYGxVxx(A/C)-	-HRDxKPxNxL-	-TxYVxTRWYRAPE(V/L)-	---
MAPKK CONSENSUS (2)	---	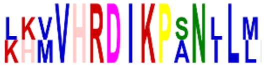	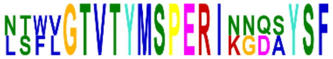	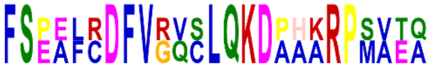
MAPKK CONSERVED	---	-VHRDIKPxNxL-	-GTVTYMSPERIxxxxYSF-	---
MAPKKK MEKK CONSENSUS (12)	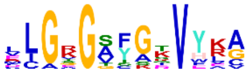	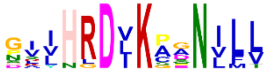	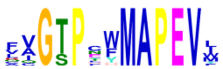	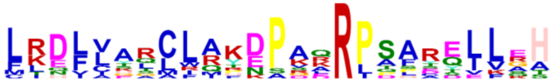
MAPKKK MEKK CONSERVED	-GxGxxGxV-	-HRDxKxxN-	-G(T/S)PxxMAPEV-	---
MAPKKK RAF A (25)	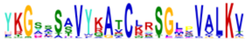	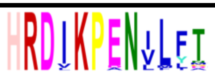	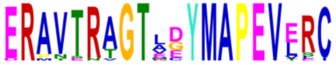	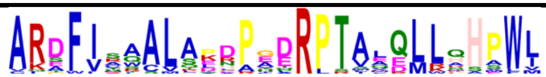
MAPKKK RAF B (7)	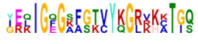 (3)	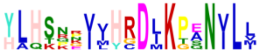	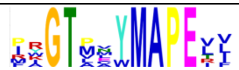	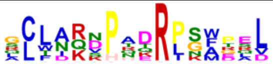
MAPKKK RAF C (7)	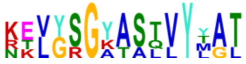	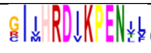 (5)<br>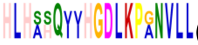 (2)	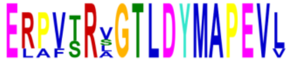	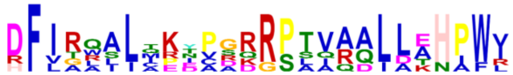

**MAPKKK RAF D (6)**	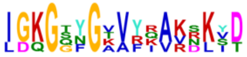	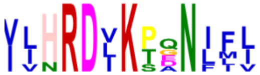	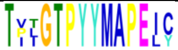 (3)<br>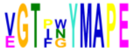 (3)	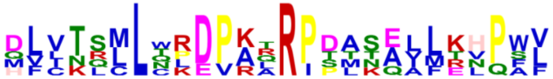
**MAPKKK RAF E (20)**	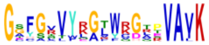 (11)	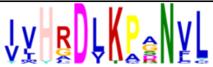	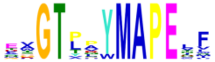	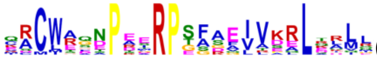 (15)
**MAPKKK RAF F (4)**	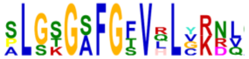	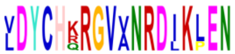	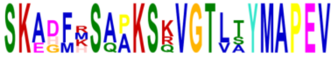	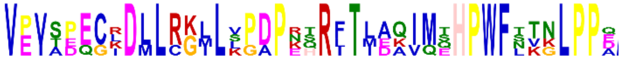
**MAPKKK RAF G (6)**	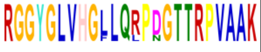	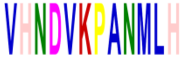	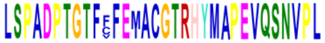	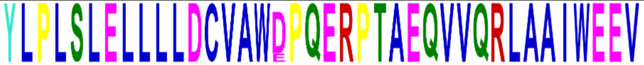
**MAPKKK RAF H (20)**	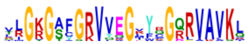	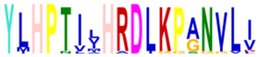	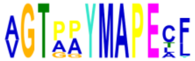	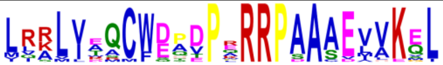
**MAPKKK RAF CONSENSUS (94)**	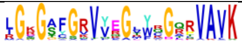<br>(32)<br>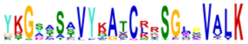<br>(26)	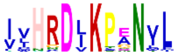 (91)	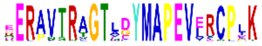 (27)<br>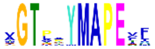 (62)	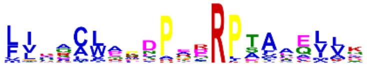 (93)
**MAPKKK RAF CONSERVED**	-GxGxxGRVxxGxxxGxxVAVK-	-HRDxKPxNxL-	-GTxxYMAPE-	---
**MAPKKK ZIK CONSENSUS**	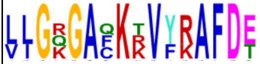	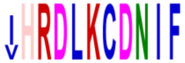	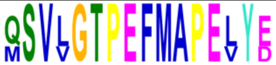	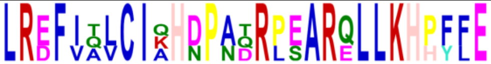
**MAPKKK ZIK CONSERVED**	-GxGAxKxVxxAFD-	-HRDLKCDNIF-	-GTPEFMAPE-	---

Table S3. Primers lists

GENE	NAME	PRIMERS	OPT.T
CreMAPK8 (*Cre01.g010000*)	RTcrMPK8-F	S: TCCCCAGTCTGCGGAAGAGCC	69 °C
	RTcrMPK8-R	AS: AAGAGGCTGCCGCAAACCAGG	
CreMAPK6 *(Cre12.g508900)*	RTcrMPK6-F	S: ATCAAGGCGATCGGCAAAGGAG	66 °C
	RTcrMPK6-R	AS: TCCGTGCGTCAATCAGGTTCTC	
CreMAPK3 *(Cre12.g509000)*	RTcrMPK3-F	S: CAACATCATCGCCGTGAAGGAC	63 °C
	RTcrMPK3-R	AS: CTGAAAGTGTTCGTTTGTGAGTGG	
CreMAPK5 *(Cre13.g607300)*	RTcrMPK5-F	S: GGCCTACGGAGTTGTATGGAAAGC	66 °C
	RTcrMPK5-R	AS: CACTTCTCGAAATGTTCGCTGTGC	
CreMAPKK1 *(Cre06.g249150)*	RTcrMEK1F3	S: CTGGCTTGGTGTCCTTCTACG	66°C
	RTcrMEK1R3	AS: GGGATCTTGCCCACCTTGG	
CreMAPKKK RAF2 *(Cre01.g003800)*	RTcr1M3K3800F	S: GCTGATGACGGCGCTGGACAT	66°C
	RTcr1M3K3800R	AS: GCGACAGACCGAAATCAGTGATCTTG	
CreMAPKKK RAF14 *(Cre02.g112500)*	RTcr2M3K2500F	S: GAATGAGGACCAGGCGCGACAGGCT	63°C
	RTcr2M3K2500R	AS: GCACCAGTCAGCCGTGAACAGG	
CreMAPKKK RAF79 *(Cre16.g674065)*	RTcr16M3K4065	S: CAGCATCTCCAGGCGCCTCTACAA	63°C
	RTcr16M3K4065	AS: GGATCTCAATCTCACGCTTGAGCATGT	

Supplemental Figures

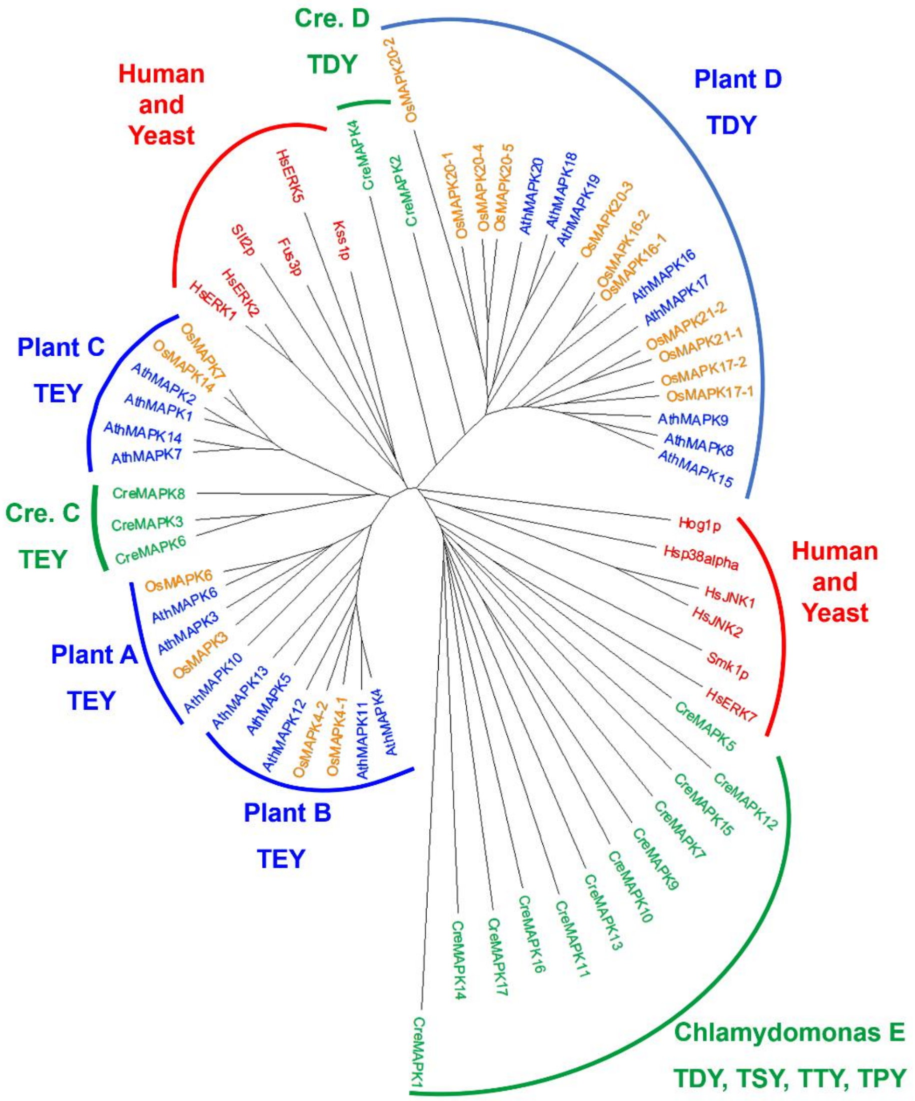

Figure S1

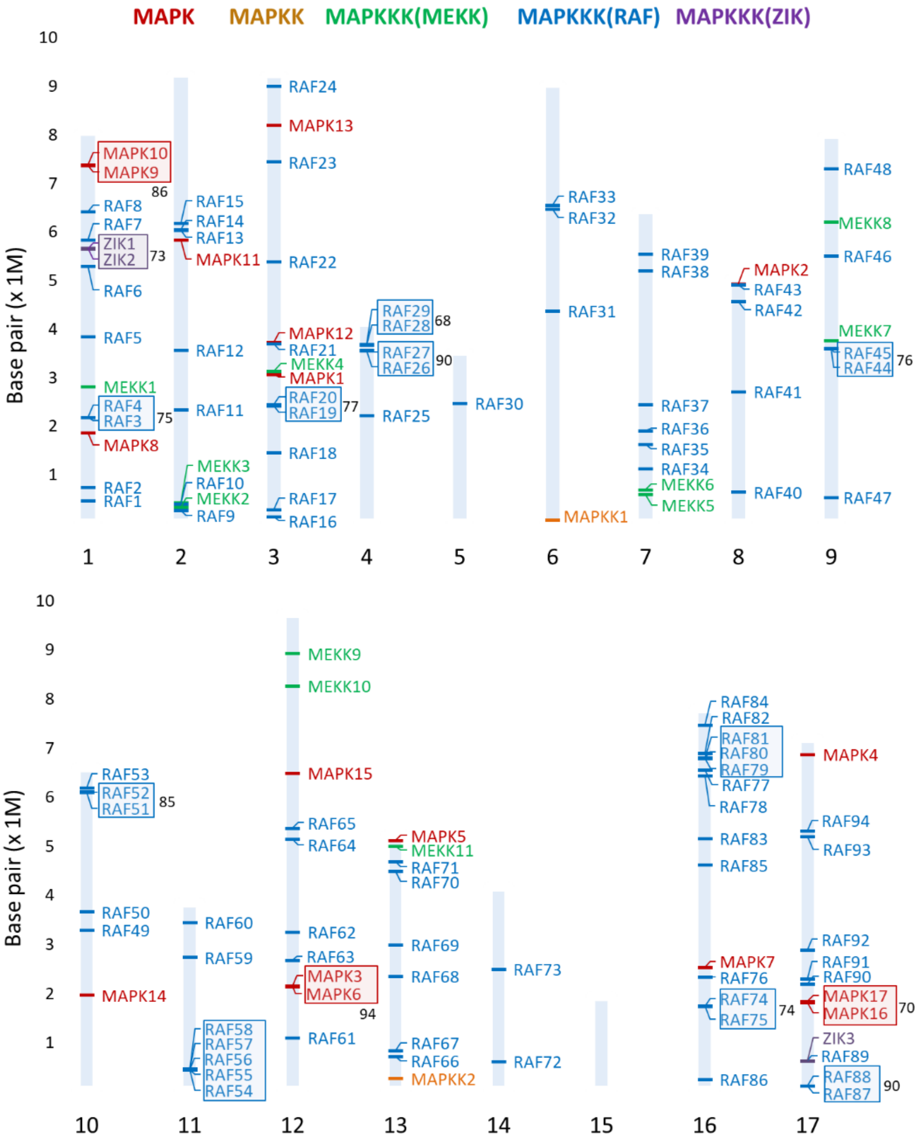
MAPK MAPKK MAPKKK(MEKK) MAPKKK(RAF) MAPKKK(ZIK)
Base pair (x 1M)
MAPK10
MAPK9
86
RAF8
RAF7
ZIK1
ZIK2
73
RAF6
RAF5
MEKK1
RAF4
RAF3
75
MAPK8
RAF2
RAF1
RAF15
RAF14
RAF13
MAPK11
RAF12
RAF11
MEKK3
RAF10
MEKK2
RAF9
RAF24
MAPK13
RAF23
RAF22
MAPK12
RAF21
MEKK4
MAPK1
RAF20
RAF19
77
RAF18
RAF17
RAF16
RAF29
RAF28
68
RAF27
RAF26
90
RAF25
RAF30
RAF33
RAF32
RAF31
MAPKK1
RAF39
RAF38
RAF37
RAF36
RAF35
RAF34
MEKK6
MEKK5
MAPK2
RAF43
RAF42
RAF41
RAF40
RAF48
MEKK8
RAF46
MEKK7
RAF45
RAF44
76
RAF47
RAF53
RAF52
RAF51
85
RAF50
RAF49
MAPK14
RAF60
RAF59
RAF58
RAF57
RAF56
RAF55
RAF54
MEKK9
MEKK10
MAPK15
RAF65
RAF64
RAF62
RAF63
MAPK3
MAPK6
94
RAF61
MAPK5
MEKK11
RAF71
RAF70
RAF69
RAF68
RAF67
RAF66
MAPKK2
RAF73
RAF72
RAF84
RAF82
RAF81
RAF80
RAF79
RAF77
RAF78
RAF83
RAF85
MAPK7
RAF76
RAF74
RAF75
74
RAF86
MAPK4
RAF94
RAF93
RAF92
RAF91
RAF90
MAPK17
MAPK16
70
ZIK3
RAF89
RAF88
RAF87
90
1 2 3 4 5 6 7 8 9
10 11 12 13 14 15 16 17

Figure S2

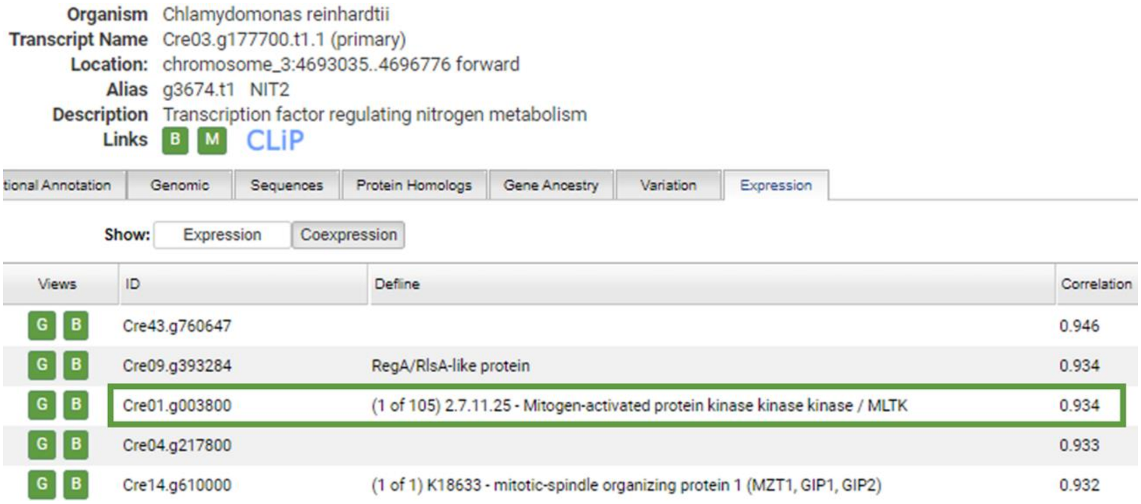
Organism Chlamydomonas reinhardtii
Transcript Name Cre03.g177700.t1.1 (primary)
Location: chromosome_3:4693035..4696776 forward
Alias g3674.t1 NIT2
Description Transcription factor regulating nitrogen metabolism
Links B M CLiP
tional Annotation
Genomic
Sequences
Protein Homologs
Gene Ancestry
Variation
Expression
Show: Expression Coexpression
Views
ID
Define
Correlation
Cre43.g760647 0.946
Cre09.g393284 RegA/RlsA-like protein 0.934
Cre01.g003800 (1 of 105) 2.7.11.25 - Mitogen-activated protein kinase kinase kinase / MLTK 0.934
Cre04.g217800 0.933
Cre14.g610000 (1 of 1) K18633 - mitotic-spindle organizing protein 1 (MZT1, GIP1, GIP2) 0.932

Figure S3
